# Multiplex Real-Time RT-PCR Assays for Detection and Differentiation of Porcine Enteric Coronaviruses

**DOI:** 10.3390/pathogens12081040

**Published:** 2023-08-14

**Authors:** Christina M. Lazov, Alice Papetti, Graham J. Belsham, Anette Bøtner, Thomas Bruun Rasmussen, Maria Beatrice Boniotti

**Affiliations:** 1 DTU Institute of Bioengineering, Technical University of Denmark, 2800 Kongens Lyngby, Denmark; cml@sund.ku.dk; 2 Istituto Zooprofilattico Sperimentale della Lombardia e dell’Emilia-Romagna, IZSLER, Reparto Tecnologie Biologiche Applicate, Via Bianchi, 9, 25124 Brescia, Italy; 3 Department of Veterinary and Animal Sciences, University of Copenhagen, 4 Stigboejlen, 1870 Frederiksberg, Denmark; 4 Department of Virus & Microbiological Special Diagnostics, Statens Serum Institut, 5 Artillerivej, 2300 Copenhagen, Denmark

**Keywords:** virus diagnosis, RNA, recombinant virus, alphacoronavirus, deltacoronavirus, porcine epidemic diarrhea virus (PEDV), transmissible gastroenteritis virus (TGEV), swine enteric coronavirus (SeCoV), porcine deltacoronavirus (PDCoV)

## Abstract

It is important to be able to detect and differentiate between distinct porcine enteric coronaviruses that can cause similar diseases. However, the existence of naturally occurring recombinant coronaviruses such as swine enteric coronavirus (SeCoV) can give misleading results with currently used diagnostic methods. Therefore, we have developed and validated three duplex real-time quantitative RT-PCR assays for the simultaneous detection of, and differentiation between, porcine epidemic diarrhea virus (PEDV) and SeCoV. Transmissible gastroenteritis virus (TGEV) is also detected by two out of these three assays. In addition, a novel triplex assay was set up that was able to detect and differentiate between these alphacoronaviruses and the porcine deltacoronavirus (PDCoV). The validated assays have low limits of detection, close to 100% efficiency, and were able to correctly identify the presence of PEDV and SeCoV in 55 field samples, whereas 20 samples of other pathogens did not give a positive result. Implementing one or more of these multiplex assays into the routine diagnostic surveillance for PEDV will ensure that the presence of SeCoV, TGEV, and PDCoV will not go unnoticed.

## 1. Introduction

Enteric pathogens contribute to significant losses in pig production worldwide; young pigs are especially vulnerable to disease caused by gastrointestinal infections [1]. Important pathogens include porcine epidemic diarrhea virus (PEDV), an *Alphacoronavirus* that caused the death of about 8 million newborn piglets within 1 year following its first introduction into the USA in 2013 [2]. Historically, PEDV is the second enteric coronavirus that has been described in pigs after the initial identification of transmissible gastroenteritis virus (TGEV). The latter was responsible for epidemic outbreaks of virus enteritis in pigs before the spike (S) protein gene deletion variant of TGEV, termed porcine respiratory coronavirus (PRCV), became widely prevalent and probably provided cross-protection against TGEV [3]. 

In recent years, new enteric porcine coronaviruses have been discovered, including swine enteric alphacoronavirus (SeACoV or swine acute diarrhea syndrome coronavirus, SADS-CoV), which has only been detected in Asia so far [4]. In addition, a porcine deltacoronavirus (PDCoV) has been identified [5]; it has been found in China, the USA, and Canada but not yet in Europe [6]. In Europe, several reports have described the detection of a diarrhea-associated, naturally occurring, recombinant virus termed swine enteric coronavirus (SeCoV) that is derived from PEDV and TGEV/PRCV [7,8,9,10,11]. The major part of the genome of SeCoV consists of a TGEV/PRCV backbone, including the nucleocapsid gene (N), whereas the S protein gene is most closely related to PEDV, with 91–92% nucleotide identity to the S gene from known PEDVs [8]. Interestingly, other recombinant PEDV strains have been characterized from Slovenia, Hungary, Italy, and Spain, where a part of the S gene (around 400 nucleotides from the 5′-end) is derived from SeCoV, whereas the rest of the genome sequence is derived from the European so-called INDEL type PEDV [10,12,13,14]. These recombinant strains seem to have outcompeted earlier European PEDV strains and have become established as the most prevalent PEDVs in Northern Italy since 2017 [12]. Similarly, in Spain, it has been found that most PEDVs detected since 2017 are of this subgroup (termed SP4) [10].

Veterinary diagnosis of enteric viral pathogens typically relies on detection of the viruses by testing nucleic acids extracted from fecal samples using real-time quantitative PCR assays [15], and, for RNA viruses, these assays include a reverse transcriptase (RT) step. Thus, the assays are commonly referred to as RT-qPCR assays. Although the clinical pictures caused by these various enteric coronaviruses associated with severe diarrhea are indistinguishable [1], typically only PEDV or TGEV would be suspected and investigated. RT-qPCR assays often used in Europe for PEDV and TGEV rely on the detection of specific targets, normally within the N or S genes [16,17]. Only after a negative result would other coronaviruses be suspected, unless prior knowledge exists about the potential involvement of other viruses, e.g., the presence of SeCoV in Italy [9]. To differentiate this recombinant virus from its parental viruses, further testing would be needed. Furthermore, it is important to maintain surveillance in order to identify the entry of other swine enteric coronaviruses such as PDCoV.

The purpose of this study was to develop easy to use duplex diagnostic assays with combined detection of both PEDV and SeCoV from suspect field samples that build on methods currently used in European veterinary diagnostic laboratories. In addition, a triplex assay that detects PDCoV, as well as the targeted porcine enteric alphacoronaviruses, has been set up and characterized. 

## 2. Materials and Methods

### 2.1. Nucleic Acids from Field Samples and Isolates

Sample materials from virus or bacterial isolates and field samples were mixed 1:10 with minimal enriched medium (MEM, internal production IZSLER, Brescia, Italy) and centrifuged for 5 min at 5000× *g* RPM at 4 °C. Sample supernatants (100 µL) and a further 100 µL of MEM plus the positive and negative controls were transferred to a sample plate and processed by the KingFisher™ Flex Purification System (ThermoFisher Scientific, Waltham, MA, USA) with the NucleoMag Vet kit (Macherey-Nagel, Düren, Germany) as previously described [16]. Extracted nucleic acid samples were stored at −80 °C. Previously extracted nucleic acids from isolates of TGEV Purdue and PEDV CV777 (IZSLER variant, GenBank accession number LT905451) were used for the primer–probe optimizations and as positive controls in the duplex RT-qPCR assays. In vitro synthesized PDCoV, TGEV, and PEDV RNA transcripts, described below, were used for the primer–probe optimizations and as positive controls in the triplex assay. Field samples were selected for use in final sensitivity testing of the multiplex assays, whereas viral and bacterial isolates were used for assessment of specificity. 

### 2.2. RNA Standards for Testing and Validation

In vitro transcribed RNA standards were generated corresponding to partial gene sequences of the S and N genes of SeCoV and PEDV, the N gene of TGEV, and the RNA-dependent RNA polymerase (RdRp) of PDCoV. In vitro transcribed RNA containing the PEDV ORF N gene was kindly provided by Beatrice Grasland, ANSES-France [18]. The PEDV S gene transcripts were produced as described [16]. Briefly, nucleic acids extracted from field samples (SeCoV strain Italy/77590/2019, accession number MT821905) or viral isolates (PEDV CV777 and TGEV Purdue) were reverse-transcribed using SuperScript IV (Invitrogen, Waltham, MA, USA, ThermoFisher Scientific) and gene-specific primers (Supplementary Table S1) according to the manufacturer’s protocol to generate cDNA. PCR amplicons of 651 bp (PEDV and SeCoV S) and 388 bp (TGEV and SeCoV N) were generated from these templates using GoTaq^®^ G2 Flexi DNA polymerase (Promega, Madison, WI, USA), gel purified, and inserted into a pCR^®^2.1-TOPO vector (Invitrogen), which was used for transformation of E.coli cells. A synthetic fragment (218 bp) corresponding to part of the PDCoV RdRp gene (KJ481931) was inserted into the plasmid pEX-A2 (Eurofins Genomics Srl, Milano, Italy) and then transferred into the pCR^®^2.1-TOPO vector as described above. Following identification of the required plasmids, linearized and purified DNAs were in vitro transcribed with HiScribe T7 RNA polymerase (New England Biolabs, Ipswich, MA, USA) according to the manufacturer’s protocol. The concentration of RNA was measured using an Infinite^®^ 200 NanoQuant spectrophotometer (Tecan, Männedorf, Switzerland), and the copy number of RNA molecules per 1 μL was calculated using the formula:

$$Copy\;number\left(\frac{RNA\;copies}{\mu l}\right)=\frac{concentration\left(\frac{ng}{\mu l}\right)}{330\frac{g}{nt}\cdot \left(nucleotides_{insert}+nucleotides_{plasmid}\right)\cdot 10^{9}\frac{ng}{g}}\cdot N_{A}$$

In this formula, the number of plasmid-derived nucleotides (nucleotides_plasmid_) in the final in vitro transcribed RNA was 128 for SeCoV N and S, whereas *N_A_* was Avogadro’s constant (approximately 6.02 × 10^23^ mol^−1^). The in vitro transcribed RNA was serially diluted with TE buffer to 10^7^ molecules/µL, aliquoted, and stored at −80 °C. 

### 2.3. Selection of Primers and Probes

Primers and probes from already established and validated assays for singleplex detection of PEDV and TGEV were selected to detect and differentiate between PEDV and SeCoV [17,19,20,21,22]. Primers and a probe for the detection of PDCoV were newly designed in this study. The primer and probe sequences are listed in Table 1. Alignments were made using partial genome sequences of PEDV and SeCoV samples tested in Italy and other publicly available sequences of these viruses. The primer and probe binding sites were then evaluated for mismatches. Three different duplex assays with different combinations of the primers and probes and one triplex assay were chosen for testing and validation. The expected properties of each assay are indicated in Table 2. 

### 2.4. RT-qPCR Protocol

The AgPath-ID One-Step RT-PCR kit (Applied Biosystems, Waltham, MA, USA, ThermoFisher Scientific) was used for all singleplex format and duplex versions of the three different duplex assays, whereas the One Step PrimeScript™ III RT-qPCR Mix (Takara Bio Inc., Kusatsu, Japan) system was used for the singleplex format and triplex versions of the triplex assays.

For the duplex assays, master mixes of 20 µL were made of primers and probes in different concentrations (see Table 3 for final concentrations); 2x RT-PCR buffer and 25x enzyme mix (Applied Biosystems) were added according to the manufacturer’s protocol, and the volume was adjusted with RNase-free water. The master mixes were aliquoted into clear plastic PCR plates and sealed with adhesive plastic foil or clear plastic strip lids. Aliquots (5 μL) of template RNA were added using a separate laboratory bench, the contents were spun down, and the PCR tubes were incubated in a CFX96 Touch Real-Time PCR Detection System (Bio-Rad Laboratories, Hercules, CA, USA). The RT-PCR program consisted of reverse transcription of RNA for 10 min at 48 °C, activation of the DNA polymerase at 95 °C for 10 min, followed by 45 amplification cycles of denaturation at 95 °C for 15 s, and annealing and elongation at 60 °C for 45 s. Fluorescence data were collected for the FAM and Cy5 channels. 

For the triplex assays, the RNA was reverse transcribed at 52 °C for 5 min, followed by Taq polymerase activation at 95 °C for 10 s. Amplification consisted of 45 cycles of 95 °C for 5 s and 60 °C for 30 s. Fluorescence data for the triplex assay were collected for the FAM, Texas Red, and Cy5 channels. 

The output files were analyzed using the program Bio-Rad CFX Maestro version 1.1 (BioRad Laboratories, Hercules, CA, USA).

### 2.5. Optimization of Primer and Probe Concentrations

Primer and probe concentrations were optimized for all singleplex assays individually by testing a few samples with a range of different primer and probe concentrations in the same run. Following the singleplex optimizations, selected samples with high and low RNA concentrations were tested in parallel in the singleplex, duplex, and triplex assays. The optimized primer and probe concentrations were used for each type of assay to assess whether the performance of the tests was similar in the multiplex and singleplex formats.

### 2.6. Standard Curves

In vitro transcribed RNA samples were 10-fold serially diluted to be in the range of 10^6^ or 10^7^ to 10^−1^ copies/5 µL, and the TGEV Purdue isolate nucleic acid extract was similarly 10-fold serially diluted (from undiluted to 10^−5^). The dilutions were tested with optimized primer and probe concentrations in three replicate samples. For the singleplex assays targeting the PEDV S and TGEV N coding sequences, in vitro transcribed RNA of SeCoV was used and for assays targeting the TGEV S coding sequence, and the RNA extracted from the TGEV Purdue isolate was used. The RNA concentration was determined by testing a 10^−4^ dilution of the RNA in the TGEV N singleplex assay together with TGEV N in vitro transcribed RNA of previously determined concentrations using the standard curve.For other assays, in vitro transcribed RNA corresponding to the relevant regions of the PEDV S and N, TGEV N, and PDCoV RdRp genes were used for testing. From the data obtained, threshold limits were decided within the log phase of amplification, and RNA standards within the linear dynamic range were selected to generate the standard curves. Standard curves were used to calculate efficiency (E = 10^−1/slope^ − 1) and RNA copy numbers in these and other runs. 

### 2.7. Validation Steps

Our aim was to validate the multiplex assays for qualitative (semi-quantitative) rather than fully quantitative use. Therefore, the assays were assessed with reference to the Minimum Information for Publication of Quantitative real-time PCR experiments (MIQE guidelines) [23] and also with reference to guidelines specific for qualitative assays [24].

RNA samples for validation were 10-fold serially diluted, as for the standard curves, but other dilutions were also used to be able to determine, more accurately, the limits of detection (LOD). The LOD were determined as the minimal number of genome copies/5 μL in six replicates for each singleplex assay and for their combinations in the multiplex assays.

Repeatability or intra-assay variation was assessed by testing three samples in six replicates of high, intermediate, and low concentrations of template, i.e., 10^6^, 10^5^/10^4^, and 10^2^ copies/5 μL for all duplex assays and 10^6^, 10^4^, and 10^3^/10^2^ copies/5 μL for the triplex assay. Concentrations were calculated for each replicate, and mean values and standard deviations were calculated from these. The coefficient of variation (CV) was obtained by expressing the relative standard deviation (standard deviation divided by the mean) as a percentage value.

Reproducibility or inter-assay variation was assessed in a similar way by testing three samples of high, intermediate, and low concentrations of the template, i.e., 10^6^, 10^4^, and 10^2^ copies/5 μL for all duplex assays and 10^6^, 10^4^, and 10^3^/10^2^ copies/5 μL for the triplex assay. The samples were tested in three replicates in four separate runs. The mean concentration was calculated for each run of each sample, and the standard deviation of the four means was calculated for each sample.

For assessment of specificity and sensitivity, single replicate testing was performed using panels of previously extracted RNA from field samples, bacteria, and virus isolates (as described below, see Supplementary Tables S2 and S3). 

## 3. Results

### 3.1. Assay Design

The primers and probes selected for use in the duplex and triplex assays for PEDV, TGEV, and PDCoV (see Table 1) were original or modified versions of oligonucleotides already in use for RT-qPCR assays for porcine coronaviruses in veterinary diagnostic laboratories [3,17,19,21,22] or newly designed oligonucleotides. The duplex and triplex combinations of these primers and probes are shown in Table 3 along with the optimal final concentrations determined experimentally. Expected characteristics of the selected assays are shown in Table 2. While each of the three duplex assays (termed Duplex 1, 2, and 3) were expected to be able to detect both PEDV and SeCoV, Duplex 3 was not expected to distinguish between them. Furthermore, TGEV should be detected by Duplex 2 and Duplex 3. The latter was intended to be specific for TGEV, whereas Duplex 2 was designed to detect PRCV as well, but without distinguishing between the two virus variants. Separately, the triplex assay was designed to detect PEDV, SeCoV, TGEV, PRCV, and PDCoV; this assay was expected to distinguish PEDV from SeCoV but without distinguishing amongst SeCoV, TGEV, and PRCV.

### 3.2. Assay Characteristics

**Figure 1:**
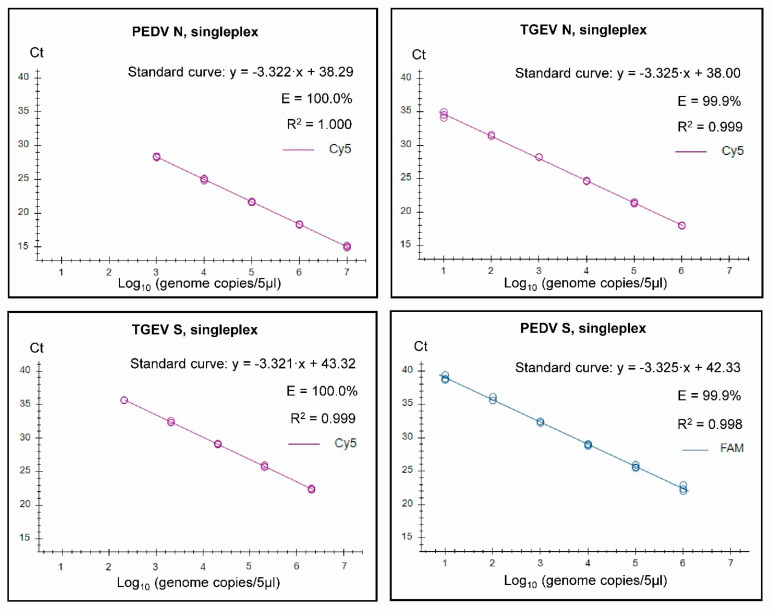
Singleplex standard curves generated on the basis of serially diluted RNA standards. In vitro transcribed RNA was used for the standards for PEDV N (from PEDV CV777), for TGEV N and for PEDV S (from SeCoV). For the TGEV S standard, RNA extracted from the virus isolate TGEV Purdue was used, and the genome copies were approximated for the run. Standard curves were generated for the data points within the linear dynamic range. Efficiency (E) was calculated automatically from the standard curve by the Bio-Rad CFX Maestro 1.1 program.

**Figure 2:**
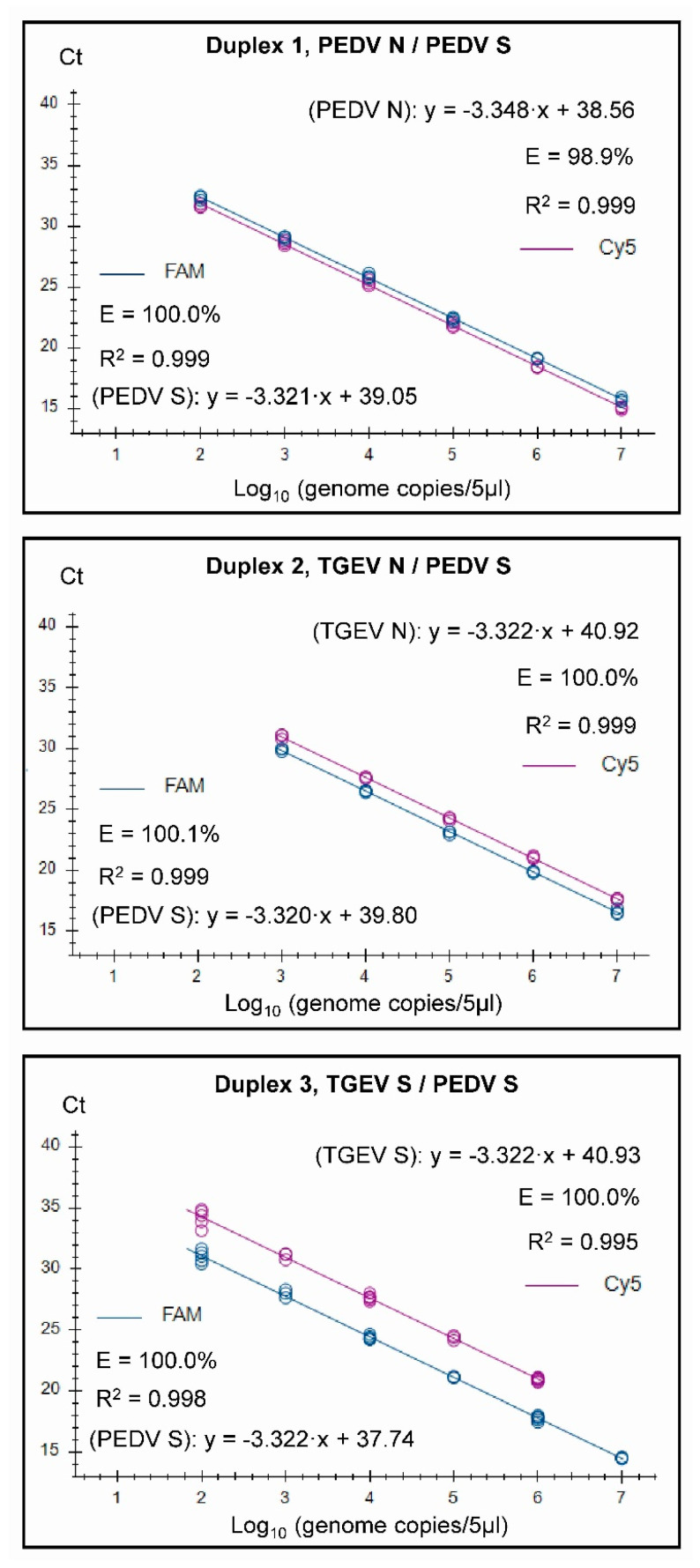
Standard curves for the three duplex assays generated on the basis of testing serially diluted RNA standards in three replicates. Each tested sample contained a mix of two standards, and fluorescence data were collected for the two channels (FAM and Cy5) as indicated.

Standard curves and derived efficiencies are shown in Figure 1 for the four individual single targets used in the three duplex assays. Similarly, the standard curves for the combined assays when run in the three duplex assays are shown in Figure 2. 

**Figure 3:**
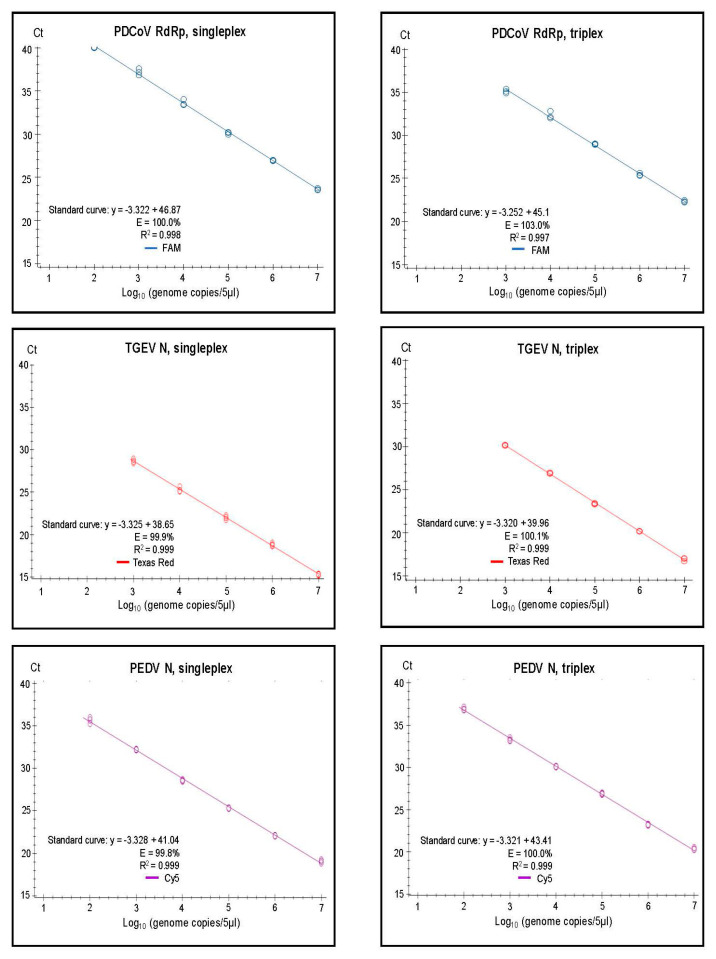
Singleplex and triplex standard curves generated on the basis of serially diluted RNA standards in three replicates. In vitro transcribed RNA was used for the standards for PDCoV RdRp, for TGEV N, and for PEDV N. Standard curves were generated for the data points within the linear dynamic range. Efficiency (E) was calculated automatically from the standard curve by the Bio-Rad CFX Maestro 1.1 program.

It was found that both the singleplex and duplex assays performed well, with close to 100% efficiencies, and the slopes of the curves were close to the optimal value of −3.32 (slope = −1/log_10_ (E + 1), i.e., when E equals 100% (or 1.00 in the formula), then the slope equals −3.32). In the Duplex 1 assay, the detection of PEDV N RNA had a slightly less than optimal efficiency of 98.9%, and a slope of −3.348 was observed. The R^2^ value was close to 1.00 for all of the assays, but, in the Duplex 3 and singleplex PEDV S assays, R^2^ was 0.998, and it was 0.995 in the Duplex 3 TGEV S. It is considered that the efficiency should preferably be in the range of 90–110%, whereas the linearity R^2^ values should be ≥0.98 [24]; thus, each of the duplex assays met these criteria. The linear dynamic ranges of the duplex assays, which were also included in the standard curves, were 10^2^ to 10^7^ RNA copies/5 µL (Figure 2), except for TGEV S in Duplex 3, where the linear range was 10^2^ to 10^6^ RNA copies/5 µL. Similarly, the RT-qPCRs in the triplex assay had a high efficiency (99.8–103%) and a R^2^ value close to 1.00 (0.997–0.999) for each target within both singleplex and triplex formats (see Figure 3). The assays had a linear dynamic range of between 10^2^–10^7^ copies for the PDCoV and PEDV (singleplex) and PEDV (triplex) assays, whereas it was between 10^3^–10^7^ copies for the TGEV (singleplex) and for PDCoV and TGEV in the triplex assays.

For the targets of the duplex assays, testing and validation showed that the LODs in the singleplex assays were in the range of 10–50 copies/5 µL, with the exception of the PEDV N assay, which gave variable and higher values for the LOD (up to 1000 copies/5 µL) (Table 4). However, the PEDV S assay had a lower LOD (just 10 copies/5 µL). In the duplex assays, consistent detection of the PEDV S gene-derived RNA down to 25 copies/5 µL template was achieved, whereas the N gene of PEDV was detected down to 50 copies/5 µL template and the TGEV S and N genes down to 100 copies/5 µL template in the three assays (Table 4). In the triplex assay, the singleplex reactions showed low LOD (10–100 copies/5 µL) (Table 4). The LOD were also low in the triplex assay for TGEV N gene (25 copies/5 µL) but higher for the PDCoV RdRp and PEDV N genes (1000 and 25 copies/5 µL, respectively).

The intra-assay variation was low (coefficient of variation 3–16%) for samples with high and intermediate concentrations of template for all the duplex and triplex assays (Table 5). However, they did not perform as well for the low concentration samples, as may be expected. Here, the coefficient of variation for detection of PEDV was 11–31% and 13–110% for TGEV. It should be noted that the concentration of this sample was close to the approximate LOD for TGEV. The inter-assay variation was not dependent on the concentration of template and showed coefficients of variation between 4 and 43% (mean 23%) (Table 5).

### 3.3. Specificity and Senstivity Testing of the Assays

Specificity testing using a panel of twenty samples of different swine pathogens (viruses and bacteria), other than PEDV, TGEV, and SeCoV (Supplementary Table S2), showed no false positive samples in any of the duplex assays. 

For sensitivity testing, a panel of fifty-five field samples of PEDV and SeCoV, including recombinant PEDV/SeCoV, as described previously [12], resulted in the expected positive tests for nearly all the samples, with just one exception (a summary of the results is shown in Table 6, whereas the full set of results, including Ct values, are in Supplementary Table S3). Out of 24 PEDV samples tested, a single PEDV sample (number 20) was not detected by either the PEDV N test in Duplex 1 or the triplex assay, but it was detected by all of the PEDV S gene tests in the Duplex 2 and Duplex 3 assays. The assays correctly differentiated between PEDV and TGEV (Duplex 2 and 3 and triplex assays). Furthermore, the assays gave distinctive patterns of results for SeCoV (negative for PEDV N and TGEV S but positive for PEDV S and TGEV N genes) compared to PEDV and the recombinant SeCoV/PEDV samples (negative for TGEV N and TGEV S but positive for PEDV N and PEDV S genes). These assays did not discriminate between PEDV and the recombinant SeCoV/PEDV genomes (Table 6, Supplementary Table S3). The triplex assay showed the same ability to detect all the tested samples except for two recombinant SeCoV/PEDV samples (numbers 25 and 42), which gave high Ct values (about 30 or above) in the duplex assays.

## 4. Discussion

The purpose of developing a duplex assay was to be able to detect both PEDVs and SeCoVs and to distinguish between them within a single assay. Different combinations of existing assays were possible, and thus three different duplex assays were designed in order to assess which assay performed the best before selecting one for diagnostic implementation. All three duplex assays included the PEDV S gene targeted assay that was originally developed after the outbreaks in the USA [16,19]. However, an assay targeting the S protein gene, which is under evolutionary selection pressure, could be challenged by accumulating genetic changes. Therefore, it is important to continue to monitor and sequence-circulating PEDV and SeCoV strains to identify mismatches in the primer/probe-binding regions of the S gene. When trying to document the absence of a pathogen, it is important to have a low limit of detection. In this study, we found that the LOD for the PEDV S gene RNA in the three duplexes were 25 copies/5 µL, which were a bit higher than in the singleplex assay (10 copies/5 µL) but still sufficient for diagnostic use. As the PEDV S assay was able to detect both SeCoV and PEDV, which were the pathogens of interest in this study, we considered that it was acceptable for the other gene targets included in the duplex assays to have higher LODs. Apart from the PEDV S target detected using the FAM-labelled probe, each of the three duplex assays in this study included one additional target detected using a Cy5-labelled probe. In Duplex 1, a PEDV N gene targeted assay was included, which was already in use for diagnostic testing of PEDV suspect samples in Denmark. When tested in the duplex format, this assay had LOD of 50 copies/5 µL, which was better than the LOD of 100 copies/5 µL observed for TGEV N and TGEV S in Duplex 2 and 3. The TGEV S of Duplex 3 was only validated using the RNA of a single TGEV isolate, and, therefore, the validation parameters, such as linear dynamic range, repeatability, and sensitivity, could possibly be improved by testing more TGEV samples and by using in vitro transcribed RNA of TGEV of known concentrations. As two of the duplex assays will detect the same target, albeit using two sets of primers and probes (PEDV in Duplex 1 and SeCoV in Duplex 2), it is possible that there will be competition for reagents in the reaction, although this did not seem to affect the performance during testing.

Overall, the assays performed well, and selection of one over the others would depend on the specific purpose, as each of the three duplex assays have their own specific properties (Table 3). Duplex 1 has a good likelihood of detecting PEDV, as two different targets were recognized. Furthermore, SeCoV was detected and distinguished from PEDV in this assay. Apart from detection of PEDV and SeCoV, Duplexes 2 and 3 were also able to detect TGEV, which could be beneficial, as this is an important differential diagnosis. However, Duplex 2 was not able to distinguish between TGEV and PRCV, which could be problematic, as PRCV is prevalent in many swine-producing countries, which are free from TGEV [3]. It should be noted, however, that although PRCV has a predominant tissue tropism for the respiratory tract, the virus is also shed via the feces [25]. This means that positive TGEV N results in Duplex 2 would have to be followed-up by further testing, e.g., using the TGEV S singleplex assay; this would enable the important discrimination between the presence of TGEV and PRCV. No testing for PRCV is necessary when using Duplex 3, but positive PEDV S results should be further examined to distinguish PEDV from SeCoV.

As previously mentioned, other porcine enteric pathogens, apart from TGEV and SeCoV, are clinically indistinguishable from PEDV. Therefore, it made sense to develop further multiplex assays capable of detecting and distinguishing between these and other relevant viruses (e.g., PDCoV and SeACoV), as shown in other studies [26,27,28]. However, none of these previously described assays specifically recognized SeCoV. 

In addition, PDCoV detection might be important, even in Europe. In fact, although PDCoV was initially identified in Asia and North America [6], it is essential to recognize that infectious diseases can cross geographical boundaries. Given the global nature of the swine industry and the extensive international trade, Europe remains susceptible to the introduction and spread of PDCoV. Integrating PDCoV detection alongside other porcine coronaviruses in diagnostic protocols enables early identification, surveillance, and monitoring of this emerging pathogen. This proactive approach provides valuable insight into the epidemiology of PDCoV, enhances preparedness for potential outbreaks, and supports the implementation of effective control measures to safeguard the European swine population. However, the triplex assay developed in this study was not able to distinguish between SeCoV and TGEV/PRCV. Further testing would be needed in cases of non-discriminative results.

It could also be worth including these pathogens into a high-throughput platform such as the Fluidigm system, using high throughput microfluidic RT-qPCRs [29], or employing several duplex or multiplex assays simultaneously, as described from Spain [30] and here. An alternative approach is to use a broad screening strategy detecting all coronaviruses, e.g., using a pan-coronavirus real-time RT-PCR assay [31,32,33,34,35], followed by further characterization of positive samples by specific RT-qPCRs or by sequencing. 

## Figures and Tables

**Table 1:** The sequences of the primers and probes used for single and multiplex assays.

	Oligo	Sequence 5′–3′	Location **	References
Duplex assays	PEDV-N-R	TTGCCTCTGTTGTTACTTGGAGAT	26,853–26,876	[17]
	PEDV-N-F	CGCAAAGACTGAACCCACTAATTT	26,679–26,702	
	PEDV-N-US-F	CGCAAAGACTGAACCCACTAACCT	26,679–26,702	[17,20]
	PEDV-N-P	TGTTGCCATT**R**CCACGACTCCTGC	26,819–26,842	
	TGEV-N-F	GCAGGTA**R**AGGTGATGTGACAA	27,637–27,658	[17]
	TGEV-N-R	ACATTCAGCCA**R**TTGTGGGTAA	27,735–27,756	
	TGEV-N-P	TGGCACTGCTCCCATTGGCAACGA	27,707–27,730	
	PEDV-S-F	ACGTCCCTTTACTTTCAATTCACA	22,474–22,497	[19]
	PEDV-S-R	TATACTTGGTACACACATCCAGAGTCA	22,559–22,585	
	PEDV-S-P	TGAGTTGATTACTGGCACGCCTAAACCAC	22,503–22,531	
	TGEV-S-F	TCTGCTGAAGGTGCTATTATATGC	20,734–20,757	[21]
	TGEV-S-R	CCACAATTTGCCTCTGAATTAGAA *	20,856–20,879	
	TGEV-S-P	* AAGGGCTCACCACCTACTACCACCA	20,764–20,788	
Triplex assay	PDCoV-RdRp-F	AACTGACATGAATGTTGGCCCT	13,777–13,798	This study
	PDCoV-RdRp-R	CATGCACCCAGAATGCGAGA	13,874–13,893	
	PDCoV-RdRp-P	AGCATACTGTGTTAGCAGAGCATGATGGT	13,815–13,843	
	TGEV-N-F	GCAGGTA**R**AGGTGATGTGACAA	27,637–27,658	[17]
	Triplex-TGEV-N-R	TGCT**R**GACACAGATGGAACACA	27,754–27,775	
	Triplex-TGEV-N-P	GGAGCAGTGCCAAGCATTACCCACAA	27,719–27,744	
	Triplex-PEDV-N-F	CGCAAAGACTGAACCCACTAAC	26,679–26,699	[22]
	Triplex-PEDV-N-R	TGGTT**R**TTGCCTCTGTTGTTACT	26,860–26,882	
	Triplex-PEDV-N-P	TGTTGCCATTGCCACGACTCCTGC	26,819–26,842	

Note: The forward and reverse primers are indicated, respectively, by the F and R suffix, whereas probes are indicated by the P suffix. The duplex assay targeting the PEDV N gene included two forward (F) primers that varied in their last three nucleotides at the 3′-end (underlined). Changes were made to some of the oligonucleotides compared to the originally published sequences: the duplex PEDV N probe (PEDV-N-P), as well as the duplex primers of the TGEV N assay, included a single degenerate base R (purine, marked in bold) corresponding to the nucleotides A and G. The forward primer of TGEV N gene was identical in both the duplex and triplex assays. The duplex PEDV-N-P was a mixture of the two already published probe sequences. The triplex PEDV R primer was modified to include genetic diversity among recently circulating PEDV strains. For the duplex and triplex TGEV N assays, these were new modifications to accommodate variations seen in recent SeCoV genomes detected in Italy. * In the TGEV S assay, a G at the 3′-end of the R primer as well as the C/T degenerate nucleotide at the 5′-end of the P were removed compared to the published assay. ** Locations of the oligonucleotides in the genome were indicated relative to reference sequences: PEDV strain CV777 (Acc. no. NC_003436), TGEV strain Purdue (Acc. no. NC_038861), and PDCoV strain USA/Illinois121/2014 (Acc. no. KJ481931), respectively.

**Table 2:** Expected characteristics of the three duplex and triplex real-time RT-PCR assays.

Expected Assay Characteristics	Duplex 1	Duplex 2	Duplex 3	Triplex
Target genes				PDCoV
	PEDV N	TGEV N	TGEV S	+
	+	+	+	TGEV N
	PEDV S	PEDV S	PEDV S	+
				PEDV N
Detects PEDV	Yes	Yes	Yes	Yes
Detects SeCoV	Yes	Yes	Yes	Yes
Detects TGEV	**No**	Yes	Yes	Yes
Detects PRCV	No	Yes	No	Yes
Detects PDCoV	No	No	No	Yes
Distinguishes PEDV from SeCoV	Yes	Yes	**No**	Yes
Distinguishes SeCoV from TGEV/PRCV	N/A	Yes	Yes	**No**
Distinguishes TGEV from PRCV	N/A	**No**	N/A	**No**

Note: Limitations of each assay are highlighted (in **bold** font) for detection and differentiation between selected porcine coronaviruses. N/A = not applicable.

**Table 3:** The conditions for the three duplex and triplex real-time RT-PCR assays, with primer and probe final concentrations and their gene targets.

Assay	Gene Target (Virus Detected)	Forward Primer	Conc.	Reverse Primer	Conc.	Probe	Conc.
Duplex 1	**PEDV N** (PEDV)	PEDV-N-F	400 nM	PEDV-N-R	800 nM	PEDV-N-probe (Cy5)	240 nM
		PEDV-N-US-F	400 nM				
	**PEDV S** (PEDV + SeCoV)	PEDV-S-F	900 nM	PEDV-S-R	900 nM	PEDV-S-probe (FAM)	200 nM
Duplex 2	**TGEV N** (TGEV/PRCV + SeCoV)	TGEV-N-F	700 nM	TGEV-N-R	700 nM	TGEV-N-probe (Cy5)	200 nM
	**PEDV S** (PEDV + SeCoV)	PEDV-S-F	900 nM	PEDV-S-R	900 nM	PEDV-S-probe (FAM)	200 nM
Duplex 3	**TGEV S** (TGEV)	TGEV-S-F	900 nM	TGEV-S-R	900 nM	TGEV-S-probe (Cy5)	200 nM
	**PEDV S** (PEDV + SeCoV)	PEDV-S-F	900 nM	PEDV-S-R	900 nM	PEDV-S-probe (FAM)	200 nM
Triplex	**PDCoV RdRp** (PDCoV)	PDCoV-RdRp-F	900 nM	PDCoV-RdRp-R	900 nM	PDCoV-RdRp-P (FAM)	200 nM
	**TGEV N** (TGEV/PRCV + SeCoV)	TGEV-N-F	900 nM	TGEV-N-R	900 nM	TGEV-N-P (Texas Red)	200 nM
	**PEDV N** (PEDV)	PEDV-N-F	700 nM	PEDV-N-R	700 nM	PEDV-N-P (Cy5)	200 nM

**Table 4:** Summary of the limits of detection (LOD) (expressed as RNA copies/5 µL) for each of the indicated assays in the singleplex and multiplex formats.

	LOD of Duplex Assays				LOD of Triplex Assay	
**Assay**	**Singleplex**	**Duplex 1**	**Duplex 2**	**Duplex 3**	**Singleplex**	**Triplex**
**PEDV N**	1000 *	50	-	-		
**PEDV S**	10	25	25	25		
**TGEV N**	25	-	100	-		
TGEV S **	50	-	-	100		
**PDCoV RdRp**					100	1000
**TGEV N**					25	25
**PEDV N**					10	25

Note: The assays were performed using the primers and probes shown in Table 1. * No consistent detection of the target in the singleplex PEDV N assay was obtained in concentrations below 1000 copies/5 µL, although individual replicates were positive in concentrations down to 200 copies/5 µL. ** Note that the TGEV LOD were determined based on an estimated concentration for the TGEV Purdue isolate RNA, as described in Materials and Methods.

**Table 5:** Summary of intra-assay and inter-assay variation.

		Tested Sample for Repeatability			Tested Sample for Reproducibility		
Assay		High Conc.	Intermediate Conc.	Low Conc.	High Conc.	Intermediate Conc.	Low Conc.
Duplex 1	**PEDV N**	4%	6%	17%	27%	27%	16%
	**PEDV S**	7%	8%	31%	29%	34%	22%
Duplex 2	**TGEV N**	4%	6%	43%	27%	27%	24%
	**PEDV S**	6%	7%	19%	29%	33%	43%
Duplex 3	**TGEV S**	7%	11%	110%	19%	21%	* 37%
	**PEDV S**	4%	3%	11%	32%	34%	35%
Triplex	**PDCoV RdRp**	10%	16%	9%	14%	16%	4%
	**TGEV N**	5%	7%	13%	10%	6%	32%
	**PEDV N**	5%	8%	22%	5%	7%	16%

Note: Intra-assay and inter-assay variation is shown as coefficient of variation (CV) from tests of three samples of high, intermediate and low concentrations (conc.) of template, i.e., 10^6^, 10^5^/10^4^ and 10^2^ copies/5 μL for the duplex assays and 10^6^, 10^4^ and 10^3^/10^2^ copies/5 μL for the triplex assay. The intra-assay variation was tested in six replicates, whereas the inter-assay was calculated in three replicates for four runs. * Five replicates were negative and excluded from the calculation.

**Table 6:** Summary of sensitivity testing for porcine alphacoronaviruses using the duplex and triplex assays.

		Duplex 1		Duplex 2		Duplex 3		Triplex		
Pathogen Samples	No. Tested	PEDV N Positive (%)	PEDV S Positive (%)	TGEV N Positive (%)	PEDV S Positive (%)	TGEV S Positive (%)	PEDV S Positive (%)	PDCoV RdRp Positive (%)	TGEV N Positive (%)	PEDV N Positive (%)
PEDV	24	23 (96)	24 (100)	0 (0)	24 (100)	0 (0)	24 (100)	0 (0)	0 (0)	23 (96)
Rec. SeCoV/PEDV	22	22 (100)	22 (100)	0 (0)	22 (100)	0 (0)	22 (100)	0 (0)	0 (0)	20 (91)
SeCoV	9	0 (0)	9 (100)	9 (100)	9 (100)	0 (0)	9 (100)	0 (0)	9 (100)	0 (0)

Note: A panel of 55 field samples of PEDV and SeCoV, including recombinant PEDV/SeCoVs, were tested in each of the multiplex assays. The number and percentages of tested samples testing positive in each assay are shown. Negative results are highlighted in grey. The full set of results (including Ct values) are shown in Supplementary Table S3.

## Data Availability

Relevant data are included in the paper.

## Supplementary Materials

The following supporting information can be downloaded at: https://www.mdpi.com/article/10.3390/pathogens12081040/s1, Table S1 [36]: Sequences of the oligonucleotides used for amplifying fragments inserted into cloning vector to enable in vitro transcription of RNA standards; Table S2: Specificity panel and results from testing in the three separate duplex assays and in the triplex assay. Table S3: Sensitivity testing for porcine alphacoronaviruses using the duplex and triplex assays.

## References

[1] Jung K., Saif L.J., Wang Q. (2020). Porcine epidemic diarrhea virus (PEDV): An update on etiology, transmission, pathogenesis, and prevention and control. Virus Res..

[2] Lee C. (2015). Porcine epidemic diarrhea virus: An emerging and re-emerging epizootic swine virus. Virol. J..

[3] Chen F., Knutson T.P., Rossow S., Saif L.J., Marthaler D.G. (2019). Decline of transmissible gastroenteritis virus and its complex evolutionary relationship with porcine respiratory coronavirus in the United States. Sci. Rep..

[4] Zhou P., Fan H., Lan T., Yang X.-L., Shi W.-F., Zhang W., Zhu Y., Zhang Y.-W., Xie Q.-M., Mani S. (2018). Fatal swine acute diarrhoea syndrome caused by an HKU2-related coronavirus of bat origin. Nature.

[5] Woo P.C.Y., Lau S.K.P., Lam C.S.F., Lau C.C.Y., Tsang A.K.L., Lau J.H.N., Bai R., Teng J.L.L., Tsang C.C.C., Wang M. (2012). Discovery of Seven Novel Mammalian and Avian Coronaviruses in the Genus Deltacoronavirus Supports Bat Coronaviruses as the Gene Source of Alphacoronavirus and Betacoronavirus and Avian Coronaviruses as the Gene Source of Gammacoronavirus and Deltacoronavi. J. Virol..

[6] Wang L., Byrum B., Zhang Y. (2014). Detection and genetic characterization of deltacoronavirus in pigs, Ohio, USA, 2014. Emerg. Infect. Dis..

[7] Akimkin V., Beer M., Blome S., Hanke D., Höper D., Jenckel M., Pohlmann A. (2016). New chimeric porcine coronavirus in Swine Feces, Germany, 2012. Emerg. Infect. Dis..

[8] Belsham G.J., Rasmussen T.B., Normann P., Vaclavek P., Strandbygaard B., Bøtner A. (2016). Characterization of a Novel Chimeric Swine Enteric Coronavirus from Diseased Pigs in Central Eastern Europe in 2016. Transbound. Emerg. Dis..

[9] Boniotti M.B., Papetti A., Lavazza A., Alborali G., Sozzi E., Chiapponi C., Faccini S., Bonilauri P., Cordioli P., Marthaler D. (2016). Porcine epidemic diarrhea virus and discovery of a recombinant swine enteric coronavirus, Italy. Emerg. Infect. Dis..

[10] de Nova P.J.G., Cortey M., Díaz I., Puente H., Rubio P., Martín M., Carvajal A. (2020). A retrospective study of porcine epidemic diarrhoea virus (PEDV) reveals the presence of swine enteric coronavirus (SeCoV) since 1993 and the recent introduction of a recombinant PEDV-SeCoV in Spain. Transbound. Emerg. Dis..

[11] Papetti A., Bonilauri P., Chiapponi C., Baioni L., Boniotti M.B. (2022). Complete Genome Sequence of an Italian Swine Enteric Coronavirus Strain 77590/2019. Microbiol. Resour. Announc..

[12] Boniotti M.B., Papetti A., Bertasio C., Giacomini E., Lazzaro M., Cerioli M., Faccini S., Bonilauri P., Vezzoli F., Lavazza A. (2018). Porcine Epidemic Diarrhoea Virus in Italy: Disease spread and the role of transportation. Transbound. Emerg. Dis..

[13] Valkó A., Biksi I., Cságola A., Tuboly T., Kiss K., Ursu K., Dán Á. (2017). Porcine epidemic diarrhoea virus with a recombinant S gene detected in Hungary, 2016. Acta Vet. Hung..

[14] Valkó A., Albert E., Cságola A., Varga T., Kiss K., Farkas R., Rónai Z., Biksi I., Dán Á. (2019). Isolation and characterisation of porcine epidemic diarrhoea virus in Hungary. Acta Vet. Hung..

[15] Mackay I.M., Arden K.E., Nitsche A. (2002). Real-time PCR in virology. Nucleic Acids Res..

[16] Bertasio C., Giacomini E., Lazzaro M., Perulli S., Papetti A., Lavazza A., Lelli D., Alborali G., Boniotti M.B. (2016). Porcine epidemic diarrhea virus shedding and antibody response in swine farms: A longitudinal study. Front. Microbiol..

[17] Kim S.H., Kim I.J., Pyo H.M., Tark D.S., Song J.Y., Hyun B.H. (2007). Multiplex real-time RT-PCR for the simultaneous detection and quantification of transmissible gastroenteritis virus and porcine epidemic diarrhea virus. J. Virol. Methods.

[18] Bigault L., Brown P., Bernard C., Blanchard Y., Grasland B. (2020). Porcine epidemic diarrhea virus: Viral RNA detection and quantification using a validated one-step real time RT-PCR. J. Virol. Methods.

[19] Alonso C., Goede D.P., Morrison R.B., Davies P.R., Rovira A., Marthaler D.G., Torremorell M. (2014). Evidence of infectivity of airborne porcine epidemic diarrhea virus and detection of airborne viral RNA at long distances from infected herds. Vet. Res..

[20] Chen Q., Li G., Stasko J., Thomas J.T., Stensland W.R., Pillatzki A.E., Gauger P.C., Schwartz K.J., Madson D., Yoon K.J. (2014). Isolation and characterization of porcine epidemic diarrhea viruses associated with the 2013 disease outbreak among swine in the united states. J. Clin. Microbiol..

[21] Vemulapalli R., Gulani J., Santrich C. (2009). A real-time TaqMan^®^ RT-PCR assay with an internal amplification control for rapid detection of transmissible gastroenteritis virus in swine fecal samples. J. Virol. Methods.

[22] Su Y., Liu Y., Chen Y., Xing G., Hao H., Wei Q., Liang Y., Xie W., Li D., Huang H. (2018). A novel duplex TaqMan probe-based real-time RT-qPCR for detecting and differentiating classical and variant porcine epidemic diarrhea viruses. Mol. Cell. Probes.

[23] Bustin S.A., Benes V., Garson J.A., Hellemans J., Huggett J., Kubista M., Mueller R., Nolan T., Pfaffl M.W., Shipley G.L. (2009). The MIQE guidelines: Minimum information for publication of quantitative real-time PCR experiments. Clin. Chem..

[24] Broeders S., Huber I., Grohmann L., Berben G., Taverniers I., Mazzara M., Roosens N., Morisset D. (2014). Guidelines for validation of qualitative real-time PCR methods. Trends Food Sci. Technol..

[25] Costantini V., Lewis P., Alsop J., Templeton C., Saif L.J. (2004). Respiratory and fecal shedding of Porcine respiratory coronavirus (PRCV) in sentinel weaned pigs and sequence of the partial S-gene of the PRCV isolates. Arch. Virol..

[26] Ding G., Fu Y., Li B., Chen J., Wang J., Yin B., Sha W., Liu G. (2020). Development of a multiplex RT-PCR for the detection of major diarrhoeal viruses in pig herds in China. Transbound. Emerg. Dis..

[27] Masuda T., Tsuchiaka S., Ashiba T., Yamasato H., Fukunari K., Omatsu T., Furuya T., Shirai J., Mizutani T., Nagai M. (2016). Development of one-step real-time reverse transcriptase-PCR-based assays for the rapid and simultaneous detection of four viruses causing porcine diarrhea. Jpn. J. Vet. Res..

[28] Si G., Niu J., Zhou X., Xie Y., Chen Z., Li G., Chen R., He D. (2021). Use of dual priming oligonucleotide system-based multiplex RT-PCR assay to detect five diarrhea viruses in pig herds in South China. AMB Express.

[29] Goecke N.B., Hjulsager C.K., Krog J.S., Skovgaard K., Larsen L.E. (2020). Development of a high-throughput real-time PCR system for detection of enzootic pathogens in pigs. J. Vet. Diagn. Investig..

[30] Puente H., Argüello H., Mencía-Ares Ó., Gómez-García M., Rubio P., Carvajal A. (2021). Detection and Genetic Diversity of Porcine Coronavirus Involved in Diarrhea Outbreaks in Spain. Front. Vet. Sci..

[31] de Souza Luna L.K., Heiser V., Regamey N., Panning M., Drexler J.F., Mulangu S., Poon L., Baumgarte S., Haijema B.J., Kaiser L. (2007). Generic Detection of Coronaviruses and Differentiation at the Prototype Strain Level by Reverse Transcription-PCR and Nonfluorescent Low-Density Microarray. J. Clin. Microbiol..

[32] Escutenaire S., Mohamed N., Isaksson M., Thorén P., Klingeborn B., Belák S., Berg M., Blomberg J. (2007). SYBR Green Real-Time Reverse Transcription-Polymerase Chain Reaction Assay for the Generic Detection of Coronaviruses. Arch. Virol..

[33] Hu H., Jung K., Wang Q., Saif L.J., Vlasova A.N. (2018). Development of a one-step RT-PCR assay for detection of pancoronaviruses (α-, β-, γ-, and δ-coronaviruses) using newly designed degenerate primers for porcine and avian fecal samples. J. Virol. Methods.

[34] Vijgen L., Moës E., Keyaerts E., Li S., Van Ranst M. (2008). A Pancoronavirus RT-PCR Assay for Detection of All Known Coronaviruses. Methods Mol. Biol..

[35] Lazov C.M., Chriél M., Baagøe H.J., Fjederholt E., Deng Y., Kooi E.A., Belsham G.J., Bøtner A., Rasmussen T.B. (2018). Detection and Characterization of Distinct Alphacoronaviruses in Five Different Bat Species in Denmark. Viruses.

[36] Kim S.Y., Song D.S., Park B.K. (2001). Differential detection of transmissible gastroenteritis virus and porcine epidemic diarrhea virus by duplex RT-PCR. J. Vet. Diagn. Investig..

