# Supplementary material for: Multiplex Real-Time RT-PCR Assays for Detection and Differentiation of Porcine Enteric Coronaviruses
Source: Pathogens. 2023 Aug 14;12(8):1040. doi: 10.3390/pathogens12081040 (PMC10457881; doi:10.3390/pathogens12081040)
Supplement: Supplementary file 1 [file pathogens-12-01040-s001.zip › Supplementary Table S1.pdf]

Supplementary Table S1

	Sequence 5′ – 3′	fragment size	Location	References
PEDV and SeCoV S	TTCTGAGTCATGAACAGCCA<br>CATATGCAGCCTGCTCTGAA	651 bp	22,103-22,122<br>22,734-22,753	[36]
TGEV and SeCoV N	CAGCAACGCTCTCGTTC<br>CTTTTGCCACTTCTGATGG	388 bp	27,541-27,557<br>27,910-27,928	This Study
PDCoV RdRp	GGAGCAGACAGGCCCGTCAGG<br>CAGGCTTTACACTTTATGCTTCCGGC	446 bp	pEX-F*<br>pEX-R**	

Sequences of the oligonucleotides used for amplifying fragments inserted into cloning vector to enable *In vitro* transcription of RNA standards. The locations of the oligonucleotides in the genome are indicated relative to reference sequences: PEDV strain CV777 (Acc. no. NC_003436) and TGEV strain Purdue (Acc. no. NC_038861). *pEX-F and **pEX-R are oligonucleotides that bind to sequences located beside the multiple cloning site of the plasmid pEX-A2.
