# Supplementary material for: Multiplex Real-Time RT-PCR Assays for Detection and Differentiation of Porcine Enteric Coronaviruses
Source: Pathogens. 2023 Aug 14;12(8):1040. doi: 10.3390/pathogens12081040 (PMC10457881; doi:10.3390/pathogens12081040)
Supplement: Supplementary file 1 [file pathogens-12-01040-s001.zip › Supplementary Table S2.pdf]

**Supplementary Table S2**

| Pathogen/Control | Duplex 1 | | Duplex 2 | | Duplex 3 | | Triplex | | |
|---|---|---|---|---|---|---|---|---|---|
| | PEDV N | PEDV S | TGEV N | PEDV S | TGEV S | PEDV S | PDCoV RdRp | TGEV N | PEDV N |
| Teschovirus | No Ct | No Ct | No Ct | No Ct | No Ct | No Ct | No Ct | No Ct | No Ct |
| Sapelovirus, TSV8 | No Ct | No Ct | No Ct | No Ct | No Ct | No Ct | No Ct | No Ct | No Ct |
| Enterovirus G | No Ct | No Ct | No Ct | No Ct | No Ct | No Ct | No Ct | No Ct | No Ct |
| Porcine circovirus 2 | No Ct | No Ct | No Ct | No Ct | No Ct | No Ct | No Ct | No Ct | No Ct |
| Hepatitis E virus | No Ct | No Ct | No Ct | No Ct | No Ct | No Ct | No Ct | No Ct | No Ct |
| *E. coli*, STb/LT | No Ct | No Ct | No Ct | No Ct | No Ct | No Ct | No Ct | No Ct | No Ct |
| *E. coli*, STb | No Ct | No Ct | No Ct | No Ct | No Ct | No Ct | No Ct | No Ct | No Ct |
| *E. coli*, STa/STb/LT | No Ct | No Ct | No Ct | No Ct | No Ct | No Ct | No Ct | No Ct | No Ct |
| *E. coli*, O8 stx2e | No Ct | No Ct | No Ct | No Ct | No Ct | No Ct | No Ct | No Ct | No Ct |
| *E. coli*, O8 | No Ct | No Ct | No Ct | No Ct | No Ct | No Ct | No Ct | No Ct | No Ct |
| *S. choleraesuis* /*S. enterica* | No Ct | No Ct | No Ct | No Ct | No Ct | No Ct | No Ct | No Ct | No Ct |
| *Cl. difficile* | No Ct | No Ct | No Ct | No Ct | No Ct | No Ct | No Ct | No Ct | No Ct |
| *Cl. perfringens* | No Ct | No Ct | No Ct | No Ct | No Ct | No Ct | No Ct | No Ct | No Ct |
| *B. pilosicoli* | No Ct | No Ct | No Ct | No Ct | No Ct | No Ct | No Ct | No Ct | No Ct |
| *B. hyodysenteriea* | No Ct | No Ct | No Ct | No Ct | No Ct | No Ct | No Ct | No Ct | No Ct |
| Rotavirus A, GF3 | No Ct | No Ct | No Ct | No Ct | No Ct | No Ct | No Ct | No Ct | No Ct |
| Rotavirus B, sample 1 | No Ct | No Ct | No Ct | No Ct | No Ct | No Ct | No Ct | No Ct | No Ct |
| Rotavirus B, sample 2 | No Ct | No Ct | No Ct | No Ct | No Ct | No Ct | No Ct | No Ct | No Ct |
| Rotavirus C | No Ct | No Ct | No Ct | No Ct | No Ct | No Ct | No Ct | No Ct | No Ct |
| Rotavirus A/B/C/H | No Ct | No Ct | No Ct | No Ct | No Ct | No Ct | No Ct | No Ct | No Ct |
| No template control | No Ct | No Ct | No Ct | No Ct | No Ct | No Ct | No Ct | No Ct | No Ct |
| TGEV Purdue isolate $10^{-3}$ | No Ct | No Ct | 26.9 | No Ct | 27.1 | No Ct | | | |
| PEDV CV777 isolate $10^{-1}$ | 28.2 | 28.1 | No Ct | 27.9 | No Ct | 28.1 | | | |
| SeCoV S and N $10^4$ copies/µl | No Ct | 26.2 | 24.1 | 26.1 | No Ct | 26.1 | | | |
| PDCoV $10^6$ copies/µl | | | | | | | 25.9 | No Ct | No Ct |
| TGEV N $10^5$ copies/µl | | | | | | | No Ct | 24.1 | No Ct |
| PEDV N $10^5$ copies/µl | | | | | | | No Ct | No Ct | 25.2 |

**Specificity panel and results from testing in the three separate duplex assays and in the triplex assay.** The samples included in the panel consisted of twenty pathogens other than PEDV, TGEV and SeCoV. The samples and the controls were tested on a single occasion. None of the samples were found positive apart from controls, when these were tested with the appropriate assays. Testing results that did not give Ct-values are indicated with grey shading.
