# Supplementary material for: Multiplex Real-Time RT-PCR Assays for Detection and Differentiation of Porcine Enteric Coronaviruses
Source: Pathogens. 2023 Aug 14;12(8):1040. doi: 10.3390/pathogens12081040 (PMC10457881; doi:10.3390/pathogens12081040)
Supplement: Supplementary file 1 [file pathogens-12-01040-s001.zip › Supplementary Table S3.pdf]

Supplementary Table S3

No.	Pathogen	Collected	Duplex 1		Duplex 2		Duplex 3		Triplex		
			PEDV N	PEDV S	TGEV N	PEDV S	TGEV S	PEDV S	PDCoV RdRp	TGEV N	PEDV N
1	PEDV	07-12-2007	21.7	21.9	No Ct	21.8	No Ct	22.5	No Ct	No Ct	21.7
2	PEDV	12-01-2019	21.0	21.2	No Ct	21.2	No Ct	21.5	No Ct	No Ct	22.9
3	PEDV	16-01-2019	21.5	21.4	No Ct	21.2	No Ct	21.8	No Ct	No Ct	23.5
4	PEDV	21-01-2019	22.0	22.8	No Ct	22.6	No Ct	23.1	No Ct	No Ct	22.6
5	PEDV	04-02-2019	16.5	17.9	No Ct	17.7	No Ct	17.9	No Ct	No Ct	17.6
6	PEDV	12-02-2019	22.8	23.5	No Ct	23.2	No Ct	23.6	No Ct	No Ct	23.3
7	PEDV	18-02-2019	19.2	20.3	No Ct	20.4	No Ct	20.4	No Ct	No Ct	20.6
8	PEDV	21-02-2019	16.0	17.0	No Ct	17.1	No Ct	17.1	No Ct	No Ct	16.1
9	PEDV	21-02-2019	18.1	18.8	No Ct	18.6	No Ct	19.0	No Ct	No Ct	18.7
10	PEDV	05-03-2019	14.9	16.6	No Ct	16.6	No Ct	16.7	No Ct	No Ct	15.7
11	PEDV	11-03-2019	21.2	23.2	No Ct	23.0	No Ct	23.2	No Ct	No Ct	22.0
12	PEDV	14-03-2019	23.7	24.7	No Ct	24.5	No Ct	24.5	No Ct	No Ct	24.5
13	PEDV	29-03-2019	22.2	22.0	No Ct	22.1	No Ct	22.1	No Ct	No Ct	23.5
14	PEDV	02-04-2019	20.5	21.9	No Ct	22.0	No Ct	22.1	No Ct	No Ct	21.3
15	PEDV	03-04-2019	19.1	20.5	No Ct	20.8	No Ct	20.8	No Ct	No Ct	20.1
16	PEDV	11-04-2019	22.4	23.6	No Ct	23.5	No Ct	24.1	No Ct	No Ct	23.4
17	PEDV	16-04-2019	25.3	25.3	No Ct	25.3	No Ct	25.8	No Ct	No Ct	26.3
18	PEDV	10-05-2019	21.5	22.6	No Ct	22.6	No Ct	23.0	No Ct	No Ct	23.4
19	PEDV	14-05-2019	15.2	17.6	No Ct	17.6	No Ct	17.7	No Ct	No Ct	16.4
20	PEDV	29-05-2019	No Ct	29.1	No Ct	29.1	No Ct	29.4	No Ct	No Ct	No Ct
21	PEDV	05-06-2019	17.5	18.9	No Ct	18.9	No Ct	19.0	No Ct	No Ct	19.2
22	PEDV	06-06-2019	15.5	17.2	No Ct	17.2	No Ct	17.2	No Ct	No Ct	17.0
23	PEDV	06-07-2019	26.8	27.7	No Ct	27.6	No Ct	27.7	No Ct	No Ct	27.7
24	PEDV	02-09-2019	15.3	17.0	No Ct	16.8	No Ct	16.8	No Ct	No Ct	16.7
25	Rec. SeCoV/PEDV	15-03-2019	31.0	31.7	No Ct	31.1	No Ct	32.1	No Ct	No Ct	No Ct
26	Rec. SeCoV/PEDV	18-03-2019	20.6	22.1	No Ct	21.9	No Ct	22.1	No Ct	No Ct	21.6
27	Rec. SeCoV/PEDV	20-03-2019	15.8	17.2	No Ct	17.1	No Ct	18.4	No Ct	No Ct	16.7
28	Rec. SeCoV/PEDV	21-03-2019	19.4	20.9	No Ct	21.1	No Ct	21.1	No Ct	No Ct	20.6
29	Rec. SeCoV/PEDV	25-03-2019	20.3	22.2	No Ct	22.3	No Ct	22.2	No Ct	No Ct	22.2
30	Rec. SeCoV/PEDV	25-03-2019	17.8	18.9	No Ct	18.4	No Ct	18.8	No Ct	No Ct	20.0
31	Rec. SeCoV/PEDV	25-03-2019	20.3	22.4	No Ct	22.1	No Ct	22.0	No Ct	No Ct	22.2
32	Rec. SeCoV/PEDV	28-03-2019	17.1	18.3	No Ct	18.3	No Ct	18.5	No Ct	No Ct	18.3
33	Rec. SeCoV/PEDV	29-03-2019	26.0	27.2	No Ct	27.2	No Ct	27.3	No Ct	No Ct	26.7
34	Rec. SeCoV/PEDV	03-04-2019	15.8	17.1	No Ct	17.0	No Ct	17.0	No Ct	No Ct	16.5
35	Rec. SeCoV/PEDV	04-04-2019	20.8	22.2	No Ct	22.1	No Ct	22.2	No Ct	No Ct	22.1
36	Rec. SeCoV/PEDV	17-04-2019	18.1	19.8	No Ct	19.6	No Ct	20.0	No Ct	No Ct	19.5
37	Rec. SeCoV/PEDV	19-04-2019	22.0	23.3	No Ct	23.2	No Ct	23.5	No Ct	No Ct	24.2

Continued Supplementary Table S3:

No.	Pathogen	Collected	Duplex 1		Duplex 2		Duplex 3		Triplex		
			PEDV N	PEDV S	TGEV N	PEDV S	TGEV S	PEDV S	PDCoV RdRp	TGEV N	PEDV N
38	Rec. SeCoV/PEDV	03-05-2019	21.5	22.8	No Ct	22.5	No Ct	22.6	No Ct	No Ct	23.0
39	Rec. SeCoV/PEDV	05-05-2019	23.2	24.4	No Ct	24.4	No Ct	24.7	No Ct	No Ct	26.0
40	Rec. SeCoV/PEDV	23-05-2019	17.7	19.1	No Ct	18.8	No Ct	19.1	No Ct	No Ct	18.4
41	Rec. SeCoV/PEDV	19-06-2019	13.0	14.2	No Ct	14.3	No Ct	14.2	No Ct	No Ct	14.3
42	Rec. SeCoV/PEDV	01-07-2019	29.9	31.1	No Ct	30.3	No Ct	31.1	No Ct	No Ct	No Ct
43	Rec. SeCoV/PEDV	24-07-2019	19.6	21.1	No Ct	21.2	No Ct	21.2	No Ct	No Ct	21.5
44	Rec. SeCoV/PEDV	06-08-2019	15.1	16.0	No Ct	15.9	No Ct	15.9	No Ct	No Ct	18.9
45	Rec. SeCoV/PEDV	19-09-2019	23.7	25.5	No Ct	25.3	No Ct	25.2	No Ct	No Ct	28.0
46	Rec. SeCoV/PEDV	20-09-2019	18.1	19.8	No Ct	19.8	No Ct	19.7	No Ct	No Ct	19.7
47	SeCoV	22-12-2009	No Ct	21.9	20.3	21.9	No Ct	21.7	No Ct	20.3	No Ct
48	SeCoV	29-12-2009	No Ct	25.1	23.2	24.6	No Ct	25.0	No Ct	24.1	No Ct
49	SeCoV	29-12-2009	No Ct	26.5	24.8	26.0	No Ct	26.3	No Ct	24.4	No Ct
50	SeCoV	29-12-2009	No Ct	22.6	21.0	22.0	No Ct	22.2	No Ct	20.4	No Ct
51	SeCoV	29-12-2009	No Ct	22.1	20.2	21.4	No Ct	21.4	No Ct	20.0	No Ct
52	SeCoV	03-02-2012	No Ct	26.2	27.1	25.9	No Ct	26.0	No Ct	26.9	No Ct
53	SeCoV	15-03-2012	No Ct	24.2	22.1	24.1	No Ct	24.1	No Ct	21.3	No Ct
54	SeCoV	27-12-2010	No Ct	22.6	21.6	22.4	No Ct	22.6	No Ct	21.6	No Ct
55	SeCoV	04-03-2019	No Ct	22.8	20.0	22.7	No Ct	23.1	No Ct	21.7	No Ct
	No template control		No Ct	No Ct	No Ct	No Ct	No Ct	No Ct	No Ct	No Ct	No Ct
	No template control		No Ct	No Ct	No Ct	No Ct	No Ct	No Ct	No Ct	No Ct	No Ct
	TGEV Purdue isolate 10^{-3}		No Ct	No Ct	26.4	No Ct	26.4	No Ct			
	TGEV Purdue isolate 10^{-3}		No Ct	No Ct	26.1	No Ct	26.5	No Ct			
	PEDV CV777 isolate 10^{-1}		27.6	28.0	No Ct	28.1	No Ct	28.4			
	PEDV CV777 isolate $10^{-1.6}$		30.2	30.8	No Ct	30.7	No Ct	31.4			
	SeCoV S and N 10^4 copies/µl		No Ct	26.1	23.4	25.8	N.T.*	N.T.*			
	SeCoV S and N 10^4 copies/µl		No Ct	26.0	23.8	26.0	No Ct	26.3			
	PDCoV 10^6 copies/µl								25.3	No Ct	No Ct
	TGEV N 10^5 copies/µl								No Ct	24.2	No Ct
	PEDV N 10^4 copies/µl								No Ct	No Ct	22.7

*N.T. = not tested

Sensitivity testing for porcine alphacoronaviruses using the duplex and triplex assays. A panel of 55 field samples of PEDV and SeCoV, including recombinant PEDV/SeCoVs, were tested in each of the multiplex assays. Ct values are shown. Testing results that did not give Ct-values are indicated with grey shading. Some of the control samples were tested in duplicate where indicated.
